# Design, preparation, and *in vitro* evaluation of gastroretentive floating matrix tablet of mitiglinide

**DOI:** 10.3389/fphar.2023.1140351

**Published:** 2023-03-15

**Authors:** Meenakshi Patel, Santosh Shelke, Naazneen Surti, Prabhakar Panzade, Lamya Ahmed Al-Keridis, Tarun Kumar Upadhyay, Nawaf Alshammari, Mohd Saeed

**Affiliations:** ^1^ Department of Pharmaceutics, School of Pharmacy, Faculty of Pharmacy, Parul University, Waghodia, India; ^2^ Department of Pharmaceutics, Srinath College of Pharmacy, Aurangabad, India; ^3^ Babaria Institute of Pharmacy, Varnama, Gujarat Technological University, Vadodara, India; ^4^ Biology Department, Faculty of Science, Princess Nourah Bint Abdulrahman University, Riyadh, Saudi Arabia; ^5^ Department of Biotechnology, Parul Institute of Applied Sciences and Centre for Research for Development, Parul University, Vadodara, India; ^6^ Department of Biology, College of Sciences, University of Hail, Hail, Saudi Arabia

**Keywords:** floating matrix tablet, mitiglinide, gastroretentive, radiography, factorial design

## Abstract

The present research is focused on developing floating matrix tablets of mitiglinide to prolong its gastric residence time for better absorption. Gastroretentive tablets were prepared using a direct compression technique with hydroxypropyl methylcellulose K15M (HPMC K15M) and sodium alginate as matrix-forming polymers and sodium bicarbonate as the gas-forming agent. A 3^2^ full factorial design was adopted to optimize the flotation and release profile of the drug. The concentration of HPMC K15M and sodium alginate were taken as the independent variables, and the floating lag time, time required for 50% drug release, and time required for 90% drug release were taken as dependent variables. The compatibility between drug and excipients was assessed by Fourier transform infrared (FTIR) spectroscopy. The prepared tablets were evaluated for different parameters such as hardness, friability, drug content, floating time, *in vitro* dissolution, and stability. Dissolution data were analyzed using various kinetic models to ascertain the mechanism of drug release. Finally, a radiographic study was conducted to estimate the retention time of the optimized floating matrix tablets of mitiglinide inside the body. The results revealed that all the physical properties of the developed formulations were within standard limits. The formulation M3, with the maximum amount of both independent variables, was considered to be the optimized formulation based on the desirability value. In addition, the optimized M3 formulation showed stability for over 6 months, as evidenced by insignificant changes in lag time, drug release pattern, and other physical properties. Furthermore, radiographic examination indicated that the tablets remained afloat in gastric fluid for up to 12 h in the rabbit’s stomach. In conclusion, the developed floating matrix tablet of mitiglinide could be regarded as a promising formulation that could release the drug in the stomach at a controlled rate and, hence, offer better management of type II diabetes.

## 1 Introduction

Oral dosage forms are popular for the delivery of medicaments, owing to their flexibility in formulation, simplicity of administration, and good patient compliance. Conventional drug delivery is linked to limitations such as insufficient bioavailability due to multiple factors (El Nabarawi et al., 2017; Laffleur and Keckeis, 2020). The fact that drugs do not stay in the stomach as long as they should for effective absorption is one such limitation. Hence, a gastroretentive floating matrix tablet can be developed to increase the drug’s residence duration in the stomach and boost bioavailability, where gastric retention time combined with longer drug release significantly improves patient compliance (Joseph et al., 2018). A floating matrix tablet is an attractive strategy to develop controlled-release formulations and provides an easy and effective way to attain a prolonged stomach residence period and sustained drug release (Yuan et al., 2023). Furthermore, controlled-release stomach-retentive dosage forms increase the bioavailability of medications with a limited window of absorption by allowing prolonged and continuous drug input to the upper gastrointestinal tract. Gastroretentive floating matrix tablets are an efficient approach to delivering certain medications with anti-diabetic effects to the upper section of the gastrointestinal tract, increasing patient compliance and providing better disease treatment (Patel et al., 2016).

Mitiglinide calcium dihydrate (MTG) is a novel insulinotropic meglitinide that particularly activates pancreatic beta cells through improved attraction and, hence, fewer side effects as compared to previous meglitinides. It addresses post-meal hyperglycemia and develops glucose regulation in the body. The mildly acidic drug MTG has a pKa value of 4.45, and hence, it stays unionized in acidic pH and acquires better absorption from the stomach. A study proved that MTG is better absorbed in the upper portion of the gastrointestinal system, and the absorption was delayed with an increase in gastric pH beyond 5 (Sorbera et al., 2000; Urashima et al., 2012). So, there are compelling reasons for creating the stomach-retentive formulation to sustain the drug concentration in the blood for better management of type II diabetic mellitus. With 1.2 h of half-life, the medication has a prompt onset and brief duration of action. Hence, it requires frequent dosing, which can be overcome by fabricating floating matrix tablets that can achieve gastroretention. The literature survey revealed that few research studies have developed the gastroretentive formulation of MTG. A group of researchers prepared the *in situ* floating gel of MTG to facilitate the sustained release of the drug in the stomach. The optimized formulation showed the *in vivo* release of the drug for over 24 h with improved bioavailability. In another study, floating microsponges of MTG were prepared using the quasi-emulsion solvent technique. The pharmacokinetic study showed an improvement in C_max_ and area under the curve with prolonged mean residence time as compared to the marketed formulation (Mahmoud et al., 2018; Mahmoud et al., 2019). In both research studies, the authors concluded that such formulations can provide better management of diabetic patients with a decreased dosing frequency. This proves the rationale for preparing the stomach-retentive sustained-release formulation of MTG.

The current exploration was undertaken to extend the gastric residence time and bioavailability of MTG for improving patient compliance in patients with type II diabetes by formulating the most acceptable solid unit dosage form, floating matrix tablets.

## 2 Materials and methods

### 2.1 Materials

Mitiglinide calcium was kindly sent by Cadila Pharmaceuticals Ltd. Ahmedabad, India. HPMC K15M and sodium alginate were purchased from Astron Chemicals, Ahmedabad. The remaining excipients were purchased from Suvidhinath Laboratories, Gujarat, India.

### 2.2 Drug–excipient compatibility study

An investigation using FTIR was conducted to determine whether the drug and excipient were compatible (Rojek and Wesolowski, 2016). The samples of the MTG-optimized floating tablet, polymers (HPMC K15M and sodium alginate), and pure drug were analyzed using FTIR. Separate mixtures of the unadulterated drug, the polymer used in the study, and the physical blend of drug and polymer were made with IR-grade KBr and scanned over a 4,000–400 cm^−1^ wavenumber range. The obtained scans were examined to determine any incompatibility between the drug and the excipients.

### 2.3 Preparation of MTG floating matrix tablets

MTG tablets of 10 mg were made using the direct compression method (Reddy et al., 2014; Rahamathulla et al., 2021). To break up lumps and achieve correct powder blending, the necessary amounts of MTG, cross-linking polymers (HPMC K15M, sodium alginate), dry binder (PVP K30), and gas-forming agent (sodium bicarbonate) were sifted *via* sieve number 80. In a mortar, the powder mixtures were correctly blended in a pattern. Magnesium stearate and microcrystalline cellulose were then added in the appropriate amounts, and the mixture was then put into a plastic bag. The double-cone blender was filled with these bags, and it was then run for 5 min. The pre-compression properties of powder mixes were assessed, and then the powder blend was compacted to prepare tablets of 5 kgcm^−2^ hardness, using 7 mm round and flat punches on a rotary tablet compression machine.

### 2.4 Factorial design

After performing an exploratory study in accordance with their earlier work on metformin, using HPMC K15M in combination with other anionic and ionic polymeric materials, the authors chose the release-controlling polymer for optimizing the formulation (Patel et al., 2017). Studies showed that sodium alginate and HPMC K15M combined as release-retarding polymers provided appropriate drug release, so these polymers were taken into consideration while creating the buoyant matrix tablet of MTG. A full factorial design was used to optimize the floating matrix tablet of MTG and investigate the impact of independent factors on the chosen dependent factors (Menon et al., 1994; Acharya et al., 2014; Narendar et al., 2016). The concentrations of HPMC K15M (X_1_) and sodium alginate (X_2_) were taken as independent factors, while dependent variables, floating lag time (Y_1_), the time required for 50% drug release (t_50_) (Y_2_), and time required for 90% drug release (t_90_) (Y_3_), were selected as responses. The concentrations of X_1_ were taken as 60 mg, 55 mg, and 50 mg as upper, medium, and low levels, respectively; for X_2_, amounts of 30 mg, 25 mg, and 20 mg were taken as upper, medium, and low levels, respectively. Design-Expert^®^ software (version 9.0.6, Stat-Ease) was used to implement and assess the design. To ascertain the impact of HPMC K15M and sodium alginate on dependent factors, the data were also subjected to a 3-D response surface approach. Table 1 provides the concentration of independent variables in the planned formulations. The amount of drug (MTG), PVP K30, sodium bicarbonate, and magnesium stearate was kept the same in all the formulations as 10 mg, 10 mg, 15 mg, and 1.5 mg, respectively. By adding an adequate amount of microcrystalline cellulose, the total weight of all the tablets was maintained at 150 mg.

**TABLE 1:** Composition of MTG floating tablets prepared by applying 3^2^ full factorial design.

**Sr No**	**Ingredients**	**Category**	**M1**	**M2**	**M3**	**M4**	**M5**	**M6**	**M7**	**M8**	**M9**	**M10**
1	MTG (mg)	Active pharmaceutical ingredient	10	10	10	10	10	10	10	10	10	10
2	PVP K30 (mg)	Binder	10	10	10	10	10	10	10	10	10	10
3	Isopropyl alcohol (mL)	Solvent for binder	q.s.	q.s.	q.s.	q.s.	q.s.	q.s.	q.s.	q.s.	q.s.	q.s.
4	HPMC K15M (mg)	Release controlling polymer	**60**	**50**	**60**	**50**	**50**	**55**	**60**	**55**	**55**	**55**
5	Sodium alginate (mg)	Release controlling polymer	**25**	**25**	**30**	**20**	**30**	**25**	**20**	**30**	**25**	**20**
6	Sodium bicarbonate (mg)	Effervescent agents	15	15	15	15	15	15	15	15	15	15
7	Magnesium stearate (mg)	Lubricant	1.5	1.5	1.5	1.5	1.5	1.5	1.5	1.5	1.5	1.5
8	Microcrystalline cellulose (mg)	Diluent to make up the weight	q.s	q.s	q.s	q.s	q.s	q.s	q.s	q.s	q.s	q.s
**Total weight per tablet (mg)**			150	150	150	150	150	150	150	150	150	150

To examine and validate the reliability of the mathematical models constructed here using full factorial design, an additional formulation suggested by the software was developed. The checkpoint batch was prepared with the levels of X_1_ (HPMC K15M) and X_2_ (sodium alginate) as −0.60 and 1, respectively. The quantities of other ingredients were kept the same as those of the batches prepared in a factorial design. The assessment of the checkpoint batch was carried out, and the outcomes from the experiments were contrasted with those that the mathematical models had anticipated. The investigational values of the responses were quantitatively matched with the expected values to authenticate the experimental design, and the relative error (%) was computed by applying Eq. 1 (Sung et al., 1996).

$$Relative\;error\;\left(\%\right)=\frac{Predicted\;value-Experimental\;value}{Predictive\;value}\;X\;100.$$ (1)

#### 2.4.1 Physical properties of floating tablets of factorial batches

The produced tablets were assessed for different post-compression evaluations, such as weight uniformity, floating lag time, hardness, drug content, friability, and tablet adhesion retention period (Srivastava et al., 2005).

#### 2.4.2 *In vitro* drug release study of factorial batches

Considering all the usual specifications in the Indian Pharmacopoeia, the drug release investigation was performed in triplicate, in a USP dissolution tester apparatus, type II (paddle method) at 37°C ± 0.5°C in 500 mL of 0.1N HCl. The amount of drug released was analyzed from the samples of the dissolving fluid; these were recorded at regular intervals by reverse-phase high-performance liquid chromatography (RP-HPLC) (Agilent Technologies 1120 series, Germany) by means of C18 Column (4.6 × 100 mm, 3.5 µ). The mobile phase was acetonitrile and water in a ratio of 55:45 (o-phosphoric acid was used to raise the pH to 2.15; the flow rate was set at 1 mL/min). The detection of eluent was carried out at 210 nm ( Sheth and Sagar, 2012). By adding fresh 0.1N HCl to the removed dissolving fluid, the sink conditions in the dissolution apparatus were maintained.

#### 2.4.3 Drug-release kinetics

Several types of kinetic models can be used to determine the drug-release pattern from the prepared batches. The *in vitro* release information of MTG from all the developed gastroretentive matrix tablets, formulated by applying a 3^2^ full factorial design, was graphed for finding the release mechanism by zero-order, Higuchi, first-order, and Korsmeyer–Peppas kinetic models. The most accurate model was deemed to have the highest correlation coefficient (Damodharan, 2020; Rehman et al., 2020).

### 2.5 Stability study

A physical stability study of the optimized formulation M3 was performed in accordance with the recommendations of the International Conference on Harmonisation (ICH) (Matthews, 1999). According to the most recent ICH guidelines, accelerated stability studies were carried out at 40°C ± 2°C and 75 ± 5% relative humidity (RH) for 6 months. After the determined period of time, the optimized tablets were scrutinized for the presence of any statistical variance in their physical characteristics, floating characteristics, and drug-release patterns (Shah, 2017).

### 2.6 *In vivo* radiographic study

The gastroretention capability of the optimized formulation could be verified using different techniques, comprising radiographic investigation, gastroscopy, gamma scintigraphy, magnetic marker monitoring, etc. (Mandal et al., 2016). Also, three fit albino rabbits weighing 2.0 kg–2.2 kg were used for the *in vivo* radiography experiments. The Institutional Animal Ethical Committee (IAEC) gave its approval to the study’s protocol (BIP/IAEC/2015/05) in accordance with its directives from the Committee for the Purpose of Control and Supervision of Experiments on Animals (CPCSEA). The X-ray opaque material was added to the optimized mixture by switching out the MTG for barium sulfate while leaving all other ingredients the same to create gastroretentive floating matrix tablets (Tadros, 2010). The content of barium sulfate in the optimized formulas was low enough to allow the formulation to float but also sufficient to make it visible by X-ray. The formulation was given to an albino rabbit after an overnight fast in order to conduct an *in vivo* X-ray imaging study. To check for radio-opaque substances in the stomach, a radiograph was obtained at 0 h, right before the tablet was administered. Water was readily available at the time of study for rabbits, but they were not permitted to consume solid food. The X-ray pictures were taken after 4 h and 12 h to track the presence of optimized floating matrix tablets in the stomach (Shin et al., 2019; Wani et al., 2020; Younis et al., 2020).

## 3 Results and discussion

### 3.1 Drug–excipient compatibility study

**FIGURE 1:**
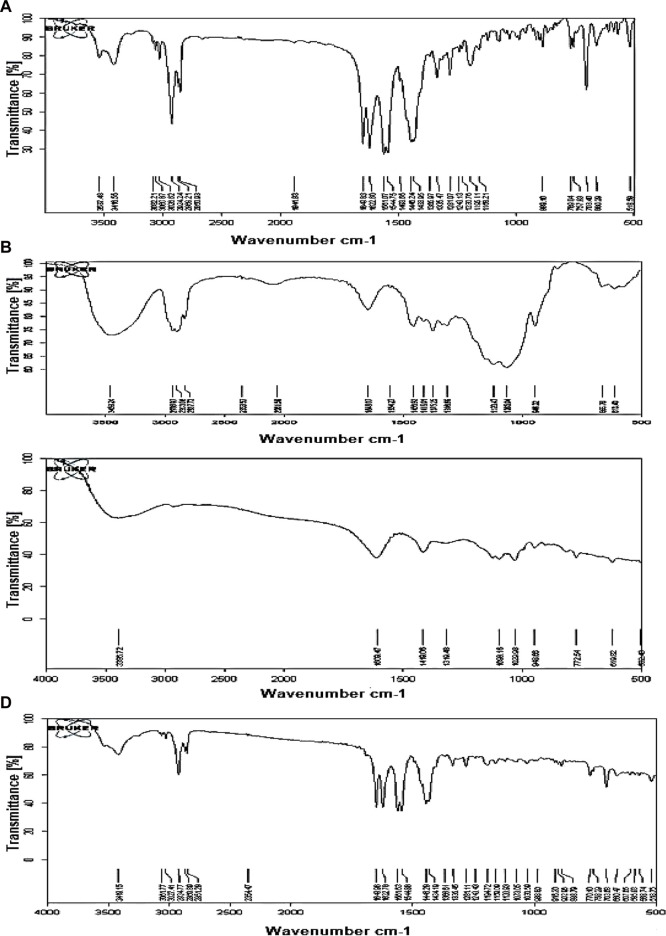
FTIR of MTG **(A)**, HPMC K15M **(B)**, sodium alginate **(C)**, and optimized formulation M3 **(D)**.

The spectra of the optimized MTG matrix tablet (M3) maintained the peaks corresponding to MTG’s distinguishing bands. This indicates the absence of any type of chemical interaction between the polymer and drug during the preparation of the formulations. The IR peaks observed for pure MTG were 3,537.48 cm^−1^ (O-H stretch); 3,416.55 cm^−1^ (N-H stretch, amide); 3,082.21, 2924.24, 2869.21, and 2,850.93 (C-H stretch); 1,649.83 cm^−1^ (C=O stretch, amide merged); 1,622.60 cm^−1^ (N-H bend); and 1,544.75 cm^−1^ (C=C stretch, aromatic). Similar peaks with a slight change in intensity were observed in the M3 formulation. The absence of drug–polymer interaction can be attributed to the lack of change and shifting of characteristic peaks of the MTG in M3 formulation. Hence, MTG is compatible with the polymer that was used to create the floating matrix tablet (Figure 1).

### 3.2 Physical evaluation of developed floating matrix tablets

All the batches of tablets that were developed fulfilled the requirements of weight uniformity evaluation. All the batches had acceptable hardness in the limit of 4.2–5.7 kg/cm^2^. The drug content of all the prepared formulations was obtained between the ranges of 99.44% and 101.97%, which is within the boundaries specified in the Indian Pharmacopeia.

The friability of all the batches was found to be less than 0.5%, which demonstrates the efficient mechanical strength of the formulations. Furthermore, the prepared batches could float for a duration greater than 12 h, and the time for the tablet’s adhesion retention was between 24.32 and 115.22 min (Table 2). It was found that as the amount of both the independent variables increased in the formulation, tablet adhesion retention time was also amplified. The tablets prepared with the minimum amount of X_1_ and X_2_ had the most direct effect on the tablet adhesion. As the polymer concentration decreased, the tablet retention period also decreased. Our findings were in accordance with the findings of Yong et al. and Derle et al., who testified that as the amount of sodium alginate and HPMC upturns in the polymeric matrix, the adhesive properties of the tablet increase (Yong et al., 2001; Derle et al., 2009).

**TABLE 2:** Physical evaluation of the designed MTG floating matrix tablets.

Batch code	Weight uniformity	Hardness (kg/cm^2^)	Drug content (%)	Friability in (%)	Floating time (hours)	Tablet adhesion retention period (min.)
M1	Complies	5.1 ± 0.28	101.92 ± 0.98	0.13 ± 0.16	>12	98.27 ± 2.43
M2	Complies	4.7 ± 0.95	100.67 ± 0.44	0.22 ± 0.17	>12	45.23 ± 3.98
M3	Complies	4.7 ± 0.03	101.32 ± 0.53	0.21 ± 0.08	>12	115.22 ± 2.54
M4	Complies	5.2 ± 0.55	100.75 ± 0.31	0.21 ± 0.54	>12	24.32 ± 3.34
M5	Complies	5.2 ± 0.15	101.97 ± 0.65	0.13 ± 0.14	>12	53.41 ± 4.92
M6	Complies	5.7 ± 0.54	100.47 ± 1.01	0.19 ± 0.11	>12	85.38 ± 4.75
M7	Complies	4.7 ± 0.38	99.83 ± 0.27	0.17 ± 0.10	>12	60.43 ± 6.21
M8	Complies	4.8 ± 0.75	99.43 ± 1.16	0.22 ± 0.08	>12	104.63 ± 4.76
M9	Complies	5.6 ± 0.85	100.18 ± 1.04	0.21 ± 0.07	>12	85.38 ± 4.75
M10	Complies	4.2 ± 0.62	100.21 ± 1.14	0.23 ± 0.12	>12	48.53 ± 5.71

^a^ n = 3, average of three determinations ± SD.

### 3.3 *In vitro* drug release study of factorial formulations

**FIGURE 2:**
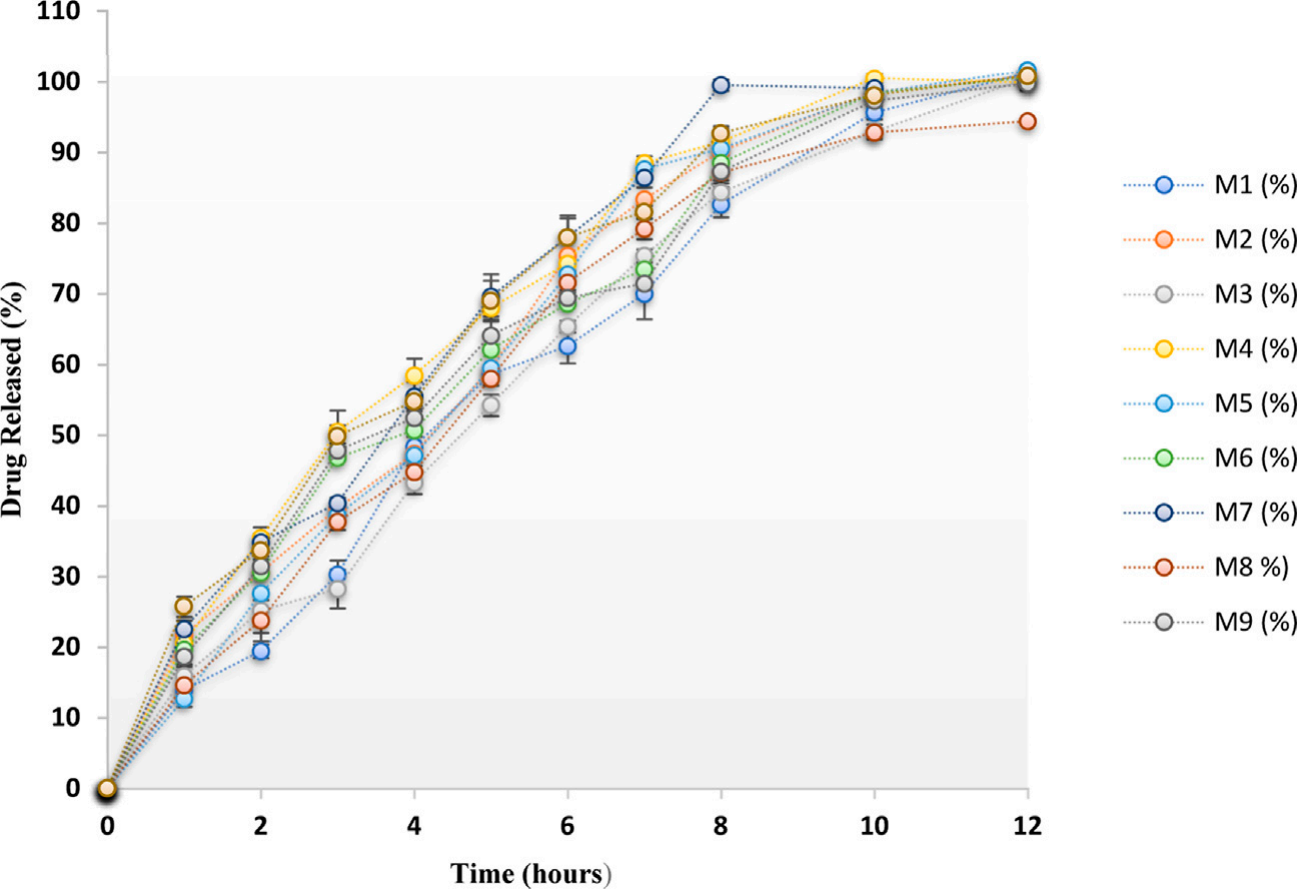
Dissolution profile of MTG floating matrix tablets prepared by a 3^2^ full factorial design.

Dissolution evaluation was performed in 500 mL of 0.1 N hydrochloric acid (HCl) as per Indian Pharmacopoeia and the release summary of the drug from all the developed preparations is presented in Figure 2. Each formulation exhibited complete drug release after 12 h. All the formulations’ drug release was sustained for 12 h, and all the formulations exhibited greater than 98% drug release except the M8 batch. The use of the proper concentrations of polymers in the tablet production process may be the cause of the drug’s prolonged release from all of the tablets.

### 3.4 Drug-release kinetic study

For the duration of 12 h, the drug discharge from the MTG floating matrix tablet formulations M4, M6, M7, M9, and M10 followed the R_HC_ model with an *R* ^2^ value near 1. The R_HC_ model’s data are derived from *in vitro* drug-release studies and are represented as the cube root of the drug concentration in the matrix formulation over time. This model is applicable to tablets whose initial geometrical properties stay constant and where dissolution occurs evenly in all planes. The rest of the formulations followed the R_0_ model with an *R* ^2^ value near 1 for a time duration of 12 h. The data are attained from *in vitro* drug-release studies and are drawn as the cumulative amount of drug released against time. This affiliation is used to explain how low-soluble drugs dissolve from matrix tablets (Chen et al., 2007). However, the *R* ^2^ value of formulation M3 was observed closer to 1, indicating zero-order drug release (Table 3).

**TABLE 3:** Results table for *in vitro* drug-release analysis by model-dependent kinetics for MTG floating matrix tablets.

Batch code	Higuchi model (RH)	Korsmeyer–Peppas model (RP)	Hixson–Crowell model (RHC)	First order (R1)	Zero order (R0)
M1	0.9469	0.9606	0.9766	0.7634	0.9809
M2	0.9627	0.9693	0.9621	0.8491	0.9776
M3	0.9466	0.9576	0.9726	0.7765	0.9845
M4	0.9774	0.9929	0.9951	0.8618	0.9637
M5	0.9505	0.9943	0.9851	0.8619	0.996
M6	0.9776	0.9892	0.9899	0.8034	0.9666
M7	0.9593	0.9766	0.9823	0.8224	0.9807
M8	0.9518	0.9912	0.9729	0.871	0.9952
M9	0.9796	0.9892	0.9905	0.8051	0.966
M10	0.9815	0.9753	0.9859	0.8736	0.9679

### 3.5 Factorial design

The full factorial analyses describe the quadratic or linear impacts of the variables on the outcome. Responses were assessed using a statistical model with interactive and polynomial terms. The polynomial equation produced under a 3^2^ full factorial design using Design-Expert software is given below as Eq. 2.

$$Y=b0+b_{1}X_{1}+b_{2}X_{2}+b_{12}X_{1}X_{2}+b_{11}X_{1}^{2}+b_{22}X_{2}^{2}.$$ (2)

Here, b_0_ denotes the intercept, b_1_ to b_22_ shows regression coefficient, and Y is the dependent variable. The usual outcome of shifting one element at a time from its bottom to top value is represented by the master effects (X_1_ and X_2_). The interaction terms are represented by X_1_ X_2_, and the quadratic effect is represented by X_1_^2^ and X_2_^2^. The effect of selected variables on responses is given in Table 4.

**TABLE 4:** Effect of independent factors on chosen responses.

Runs	Batch code	Floating lag time (sec)	Time required for 50% (t_50_) (hours)	Time required for 90% (t_90_) (hours)
1	M1	35.46 ± 2.43	4.14 ± 0.26	9.42 ± 1.01
2	M2	45.41 ± 3.21	4.23 ± 0.31	7.99 ± 0.81
3	M3	10.33 ± 1.91	4.63 ± 0.42	9.69 ± 0.91
4	M4	35.12 ± 2.19	3.01 ± 0.18	7.87 ± 0.43
5	M5	40.18 ± 3.28	4.25 ± 0.29	7.96 ± 0.66
6	M6	10.23 ± 0.93	3.95 ± 0.37	9.1 ± 0.37
7	M7	33.54 ± 2.43	3.6 ± 0.15	7.24 ± 0.52
8	M8	6.83 ± 0.79	4.47 ± 0.25	9.71 ± 1.01
9	M9	11.28 ± 1.02	3.82 ± 0.27	9.2 ± 0.45
10	M10	15.41 ± 1.73	3.65 ± 0.19	7.77 ± 0.42

^a^ n = 3, average of three determinations ± SD.

**FIGURE 3:**
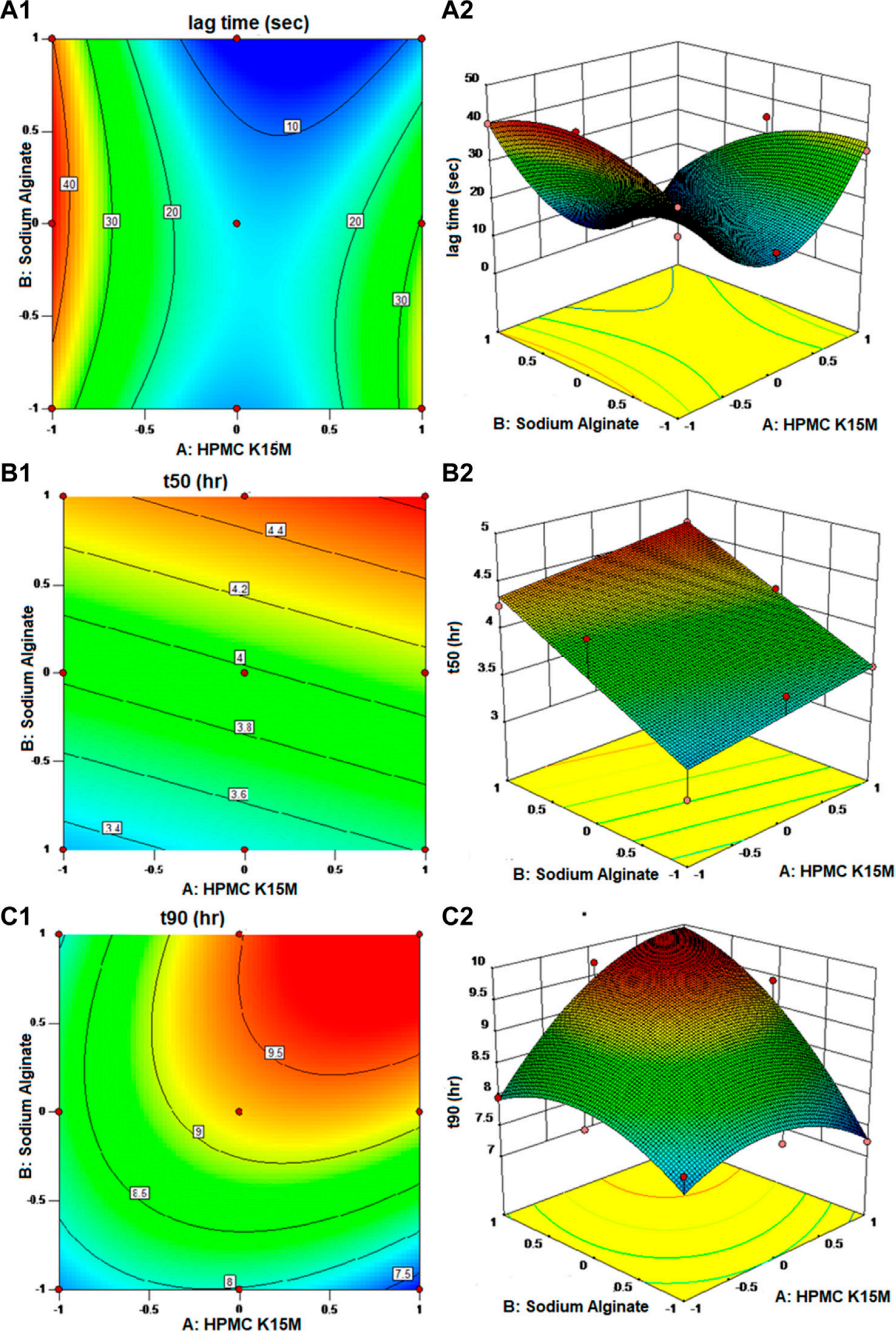
Contour plot and response surface plot: **(A1, A2)** lag time, **(B1, B2)** time to release 50% of the drug, and **(C1, C2)** time to release 90% of the drug.

Entire developed formulations produced acceptable floating lag times between 6.83 and 45.41 s, proving that the designated independent factors had no discernible influence upon the dependent factors. Nearly 90% of the medication was released by the formulations between 7.24 and 9.71 h, and 50% was released between 3.01 and 4.47 h. The surface response plot and contour plot for each reaction are shown in Figure 3.

The *p* values for F_lag_, t_50_, and t_90_ were found to be 0.0230, 0.0039, and 0.0430, respectively, which are less than 0.0500, demonstrating the significance of model terms. The quadratic model was observed to be important for floating lag time (F_lag_) and time to release 90% of the drug (t_90_), whereas the response and time to release 50% of the drug (t_50_) followed the linear model. The excellent correlation coefficients for F_lag_ (*R* ^2^ = 0.9634), t_50_ (*R* ^2^ = 0.8420), and t_90_ (*R* ^2^ = 0.9437) demonstrated a superior fit.

The floating lag time ranged from 6.83 to 45.42 s. *R* ^2^ was observed to be equal to 0.9634. The model appears to be significant because the F-value was found to be 15.80. Only 2.30% of the time is it possible for noise to cause an F-value this large. The following Eq. 3 is the fitted equation for the responses:

$$F_{lag}=+14.89-7.00X_{1}-4.50X_{2}-7.00X_{1}X_{2}+22.67X_{1}^{2}-6.83X_{2}^{2},$$ (3)

It is clear from the abovementioned equation that sodium alginate and the quantity of HPMC K15M both have a negative impact on the floating lag time of the developed floating matrix tablets. This may be credited to the gelling competency of both polymers, which makes the matrix formulation dense. HPMC retards the release by controlling the diffusion and erosion of the matrix, and sodium alginate is a natural polysaccharide that forms a gel in the presence of calcium. As soon as sodium alginate comes into contact with calcium ions, the sodium ions (Na^+^) exchange for the calcium ions (Ca^2+^), and the polymers become cross-linked, resulting in the formation of a gel (Nokhodchi and Tailor, 2004; Bera et al., 2016). The same mechanism is used in the preparation of sodium alginate beads (Bangun et al., 2021). As a result, as the level of both factors elevates, the floating lag time declines, which is desirable. Contour plots were used to clarify the association between the independent and dependent factors. The plots were curvy, signifying a non-linear association between X_1_ and X_2,_ and the quadratic effect of the HPMC K15M was also spotted.

In the case of the time required to release 50% of the drug (t_50_) response, the *p* values for factors X_1_ and X_2_ were found to be 0.1725 and 0.0016, respectively, indicating that only X_2_ has a significant effect on the time required to release 50% of the drug from the formulation. As the response, t_50_ followed a linear model; there was no interaction or quadratic effect observed between the independent factors on the response.

$$t_{50}=+3.98+0.15X_{1}+0.52X_{2},$$ (4)

Equation 4 shows that both factors, the amount of HPMC K15M (X_1_) and sodium alginate (X_2_), have a positive effect on t_50_ of the formulated floating matrix tablets of MTG, which signifies that as the absorption of both variables increases, the t_50_ increases. This might be explained by the fact that both polymers have a characteristic that suspends the release of the MTG from the matrix system. This effect obtained by sodium alginate on t_50_ was contradictory to the findings of Prajapati et al. (2009). This is probably because of the fact that the drug is in its salt form. The sodium alginate interacts with the calcium ions of the salt form of the drug, thus enhancing the gelling property of sodium alginate. The effect obtained by HPMC K15M was in accordance with the earlier findings, where the increased amount of HPMC increased the time to release 50% of the drug out of the formulation (Singh and Saini, 2016). The plots were flat, demonstrating a linear association between X_1_ and X_2_ with no interactions between the variables.

Similarly, the *p* values for factors X_1_ and X_2_ were found to be 0.0680 and 0.0158, respectively, indicating that only X_2_ has significance in the model. The interaction effect was found significant, and the quadratic effect of X_1_ and X_2_ were observed to be insignificant.

$$t_{90}=+9.22+0.42X_{1}+0.75X_{2}+0.59X_{1}X_{2}-0.53X_{1}^{2}-0.50X_{2}^{2},$$ (5)

As shown in Eq. 5, the amounts of HPMC K15M (X_1_) and sodium alginate (X_2_) have agonistic effects on the t_90_ of the formulated floating matrix tablets of MTG, which means that as the concentrations of both variables increase, the t_90_ also increases. Additionally, the observation was that the positive effect of X_2_ was more significant than that of X_1_. The interaction effect between X_1_ and X_2_ was highly substantial, with an agonistic effect on the response. A quadratic effect of both the independent variables had an antagonistic effect on the time it took to release 90% of the drug. This indicates that the ideal concentrations of X lie within the experimental region rather than at its edges. These impacts were further exemplified in contour and surface response plots. The curvy graphs disclosed a non-linear affiliation between X_1_ and X_2_. It was evident that t_90_ was near 9.5 h with the maximum amounts of both variables (Timmins et al., 1997; Prajapati et al., 2008).

### 3.6 3^2^ Full factorial experimental design: Validation study

The checkpoint batch was formulated with the levels of X_1_ (HPMC K15M) and X_2_ (sodium alginate) as −0.60 and 1, respectively. The predicted values of responses for the checkpoint batch, given by Design-Expert software, were 12.9207 s for floating lag time, 4.41 h for t_50,_ and 8.68 h for t_90_. The batch was formulated and evaluated to get the actual values of the responses. The experimental response values for the formulated batch were determined to be 13.21 s for floating lag time, 4.21 h for t_50_, and 8.38 h for t_90_. The comparative errors (%) amongst the practical and predicted values for each response were found to be 2.23% (F_lag_), 4.436% (t_50_), and 3.498% (t_90_), which were all within 5%. This shows that there was agreement between the practical and expected results, demonstrating the predictability and validity of the model. By applying limits to both the responses of the dependent variable and the independent factors, the optimal preparation was produced. The controls for the responses, floating lag time, t_50_, and t_90_, were set to a minimum, between 4–5 h and 8–10 h, respectively.

**FIGURE 4:**
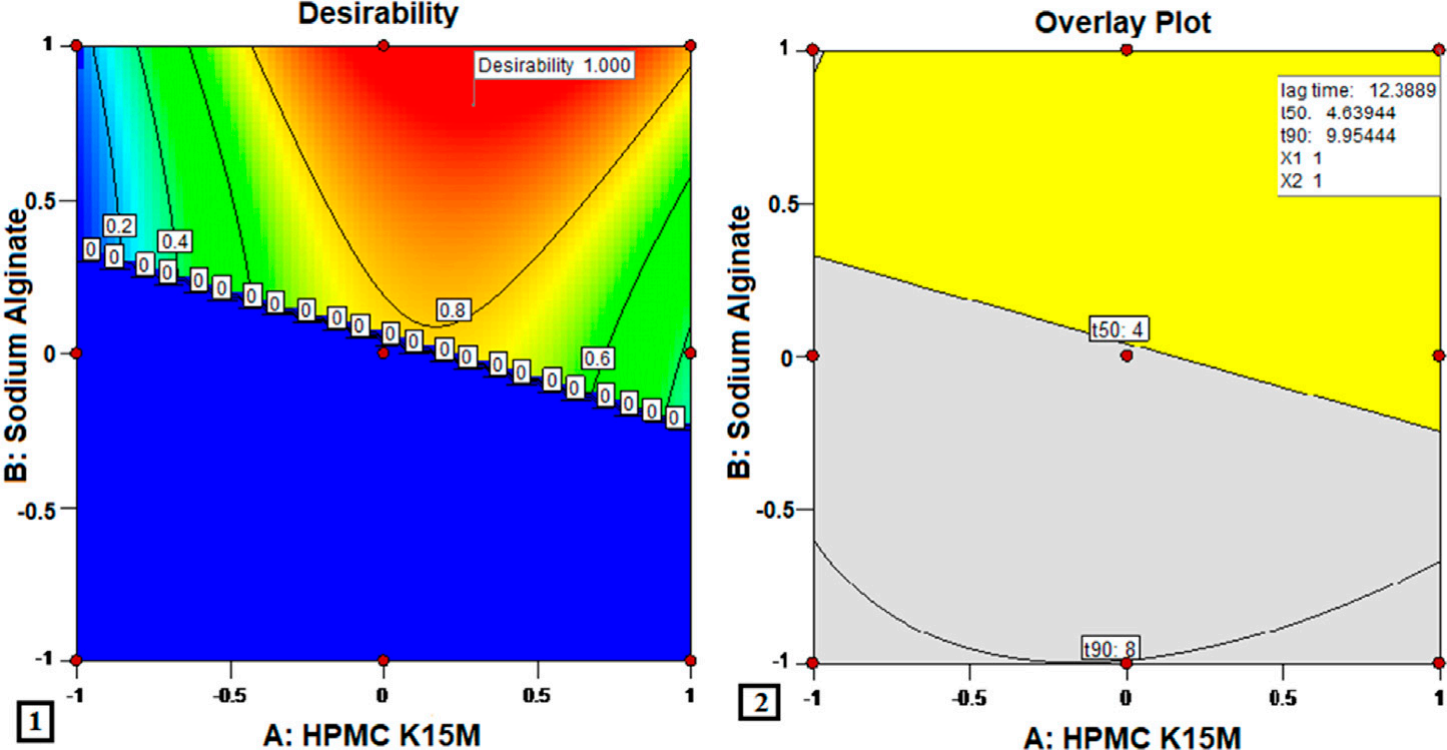
Optimization of MTG floating matrix tablet (1) desirability function and (2) overlay plot.

Design-Expert software used the plots with the highest desirability, close to 1.0, to determine the suggested concentrations of the independent variables. The optimized region for getting the desired values of responses was obtained in the complete range of X_1_ and between the ranges of 0.3 and 1 of X_2_. As a result, formulation M3, having a desirability of 1, and lying in the yellow area of the overlay graph, was deemed to be the optimized formulation. Figure 4 shows the optimized formulation with the level of X_1_ and X_2_ equal to 1, with the predicted responses of 12.38 s, 4.63 h, and 9.954 h for the floating lag times, t_50_, and t_90_, respectively. The observed values of floating lag time (10.33 ± 0.91), t_50_ (4.63 ± 0.42), and t_90_ (9.69 ± 1.91) were quite similar to the values predicted by the model.

### 3.7 Stability study of optimized formulation

The results obtained after 3 and 6 months of an accelerated stability study of an optimized gastroretentive floating matrix tablet (M3) exhibited no substantial changes in the physical properties and buoyancy parameters. The drug-release pattern of the optimized matrix tablet had 90% similarity with the formulation after 3 and 6 months of the stability study. The variation in the release pattern was insignificant. Hence, it can be claimed that formulation M3 has adequate stability during storage at 40°C under 75% RH for six months (Nayak and Pal, 2011).

### 3.8 Radiographic study

**FIGURE 5:**
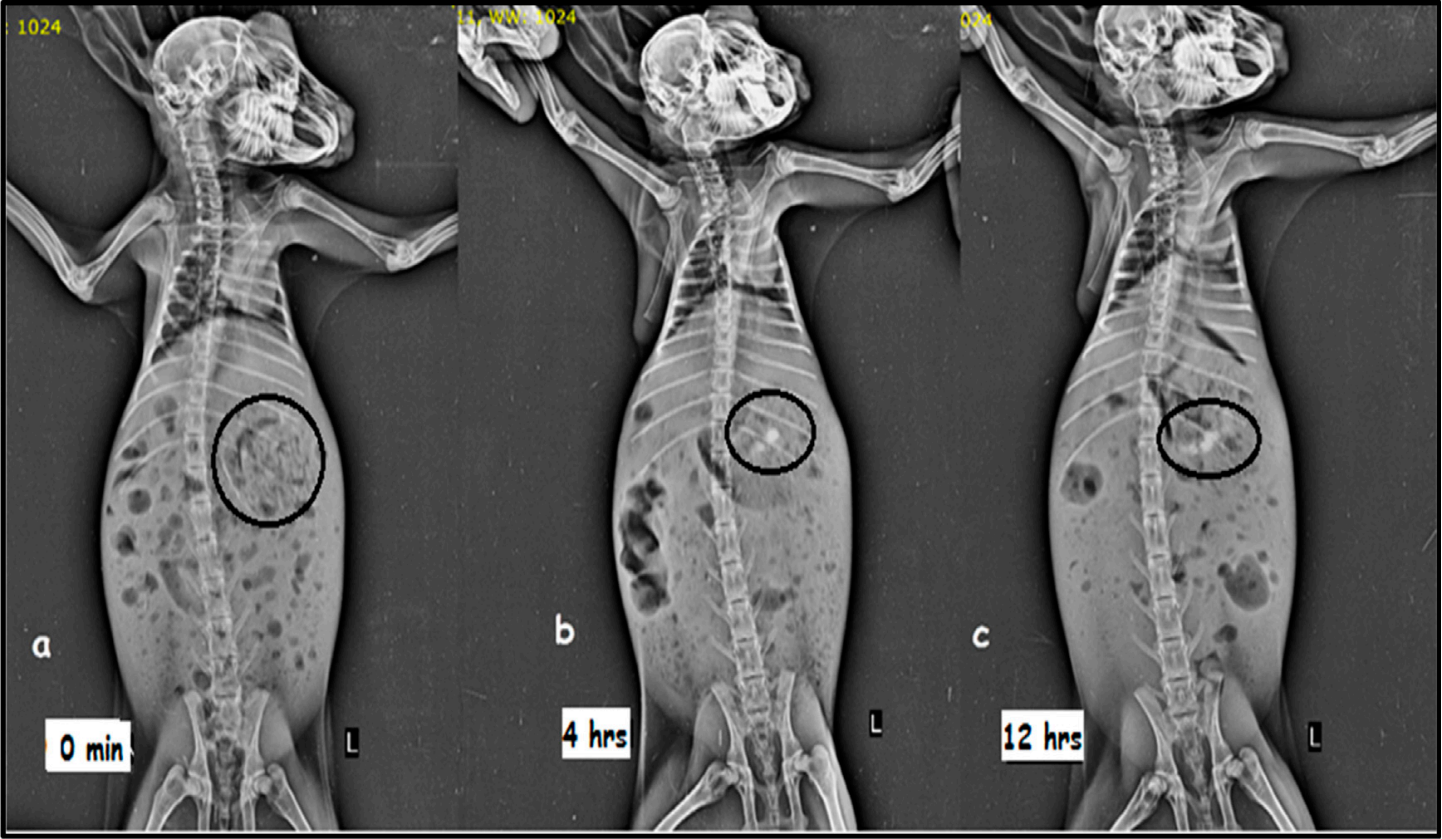
X-ray images showing the presence of barium sulfate-loaded floating matrix tablet in the rabbit’s stomach: **(A)** 0 min, **(B)** 4 h, and **(C)** 12 h.

Figure 5 shows the X-ray images obtained at 0, 4, and 12 h. The pictures evidently show white spots in the stomach, which indicates that the formulation stayed buoyant in the rabbit stomach for 12 h in gastric fluid. Thus, the study supports the floating matrix tablet for MTG’s gastroretentive activity (Patel M et al., 2021; Patel MB et al., 2021).

## 4 Conclusion

The research concludes the successful preparation of floating matrix tablets of MTG by applying a 3^2^ full factorial design using polymers HPMC K15M and sodium alginate as independent factors. The formulations were formulated by the direct compression method, and they exhibited buoyancy for 12 h. Results showed that the M3 formulation, which had the highest concentration of both factors, released MTG for a 12-h period and fell in the yellow zone of the overlay graph, which was regarded as the optimal formulation. The optimized formulation’s gastroretention was confirmed by a radiographic analysis of the barium sulfate-loaded tablets. Therefore, the developed gastroretentive matrix tablets reduce the dosing frequency of MTG with enhanced bioavailability and diminished side effects.

## Data Availability

The raw data supporting the conclusions of this article will be made available by the authors, without undue reservation.
